# Predicting postprandial glucose excursions to personalize dietary interventions for type-2 diabetes management

**DOI:** 10.1038/s41598-025-08003-4

**Published:** 2025-07-17

**Authors:** Victoria Brügger, Tobias Kowatsch, Mia Jovanova

**Affiliations:** 1 Centre for Digital Health Interventions, School of Medicine, University of St. Gallen, St. Gallen, Switzerland; 2 Institute for Implementation Science in Health Care, University of Zurich, Zurich, Switzerland; 3 Centre for Digital Health Interventions, Department of Management, Technology and Economics, ETH Zurich, Zurich, Switzerland

**Keywords:** Type-2 diabetes management, Machine learning, Dietary interventions, Personalized nutrition, Just-in-time-adaptive interventions (JITAIs), Nutrition, Health care, Nutrition

## Abstract

Elevated postprandial glucose levels present a global epidemic and a major challenge in type-2 diabetes (T2D) management. A key barrier to developing effective dietary interventions for T2D management is the wide inter-individual variation in glycemic and behavioral responses, which limits the impact of one-size-fits-all recommendations. To enable personalized dietary prompts for glycemic control, it is critical to first predict an individual’s susceptibility to elevated postprandial (PPG) levels—or state of momentary vulnerability to PPG excursions. We examined the feasibility of personalized models to predict PPG excursions,and their associated vulnerability states, in the daily lives of 67 Chinese adults with T2D (*M*age = 61.39; median = 63.00; 35 women; 2,463 glucose observations). We developed machine learning models trained on past individual observations to predict the *next-in-time* PPG excursion, using continuous glucose monitoring (CGM) data or CGM data combined with manually-logged meals and glucose-lowering agent intake. On average, personalized models predicted PPG excursions (F1-score: *M* = 75.88%; median = 78.26%), with substantial variation in predictability across individuals. Notably, no two individuals shared the same dietary and temporal predictors of PPG excursions. This study is the first to predict individual vulnerability states to glucose responses among adults with T2D in China. Findings can help personalize just-in-time adaptive interventions by tailoring dietary prompts based on individuals’ unique vulnerability states to PPG excursions. This approach can inform the development of digital dietary interventions in mHealth apps and clinical decision support tools, thereby helping optimize glycemic control and patient-centered T2D lifestyle management..

**Supplementary Information:**

The online version contains supplementary material available at 10.1038/s41598-025-08003-4.

## Introduction

Type-2 diabetes (T2D) accounts for more than 90% of the 537 million diabetes cases worldwide^1^ and this number is projected to reach at least 1.3 billion by 2050^2,3^. Given its growing global burden, effective management strategies are urgently needed. Nutrition plays a central role in T2D management^4^ with dietary recommendations—such as adopting low-calorie^5,6^, low-carbohydrate^7^, and low glycemic index diets^8^—often recommended to control blood glucose levels^9,10^.

Postprandial glucose (PPG) excursions, often characterized as post-meal spikes in blood glucose levels, present one key target for dietary interventions in T2D management^11^. PPG excursions contribute to elevated glycated hemoglobin (HbA1c)—a long-term marker of blood glucose^12^—and are independently associated with an increased risk of cardiovascular complications^13,14^, neuropathy^15^, and kidney damage^15^. PPG levels exhibit both inter- and intra-individual variability across relatively short time frames, related to meal composition (fat, carbohydrates, protein, fiber), meal timing, as well as lifestyle factors, such as stress, sleep, and physical activity^16–18^, among other factors. PPG excursions can be measured using continuous glucose monitors (CGMs), which insert a small needle under the skin (usually on the abdomen or upper arm) and track glucose levels in the interstitial fluid—typically every 5 to 15 minutes^19^. Together, the near-real-time monitoring provided by CGMs and high temporal variability, make PPG excursions a modifiable and actionable target for dietary interventions in real-world, non-clinical settings^20^.

However, a key challenge for dietary interventions that aim to predict and prevent PPG excursions is the substantial inter-individual variability in both glycemic and behavioral responses. Individuals with T2D show different post-meal glucose patterns to the same foods and vary in adherence to dietary recommendations. General guidelines often recommend reducing carbohydrate intake or increasing fiber, but these strategies may not be equally effective for all individuals^9,10,21^. CGM-based studies have shown that two individuals can exhibit different glycemic responses to the same carb-standardized meals^22,23^, suggesting that generic recommendations (e.g.,  lower carb intake) may not have uniform effects across individuals. This inter-individual variability has been linked, in part, to differences in gut microbiota and genetic factors^23–25^. Beyond biological differences, variability in the effectiveness of dietary recommendations is further compounded by a combination of psychological, behavioral, and socio-economic factors that can influence individuals’ adherence to recommendations and overall T2D self-management^26–30^. Together, these individual differences underscore limitations of one-size-fits-all dietary approaches and call for more targeted dietary interventions.

A growing body of work points to a shift toward personalized nutrition—that is, personalizing dietary recommendations and nutrition plans to an individual’s unique characteristics, such as their biology, lifestyle, health status, environment, and preferences^31,32^ ,with increasing evidence that personalized dietary interventions can improve cardiometabolic health outcomes, relative to standardized dietary recommendations^33,34^. Advances in digital health technologies^35^ including just-in-time adaptive interventions (JITAIs), offer new opportunities to deliver such personalized interventions as individuals go about their daily lives^36^. JITAIs can integrate continuous data from smartphones and wearables (e.g., CGMs) to dynamically adapt and deliver context-sensitive dietary prompts. JITAIs have shown promise for behavior change in smoking cessation^37,38^ and physical activity^39^ but their application for T2D dietary management is still emerging^40,41^. A critical first step in developing JITAIs for glycemic control is to examine whether PPG excursions can be predicted at the individual level and identify which factors (e.g., food choices and meal timing) are most predictive of an individual’s state of vulnerability^36^—period of heightened susceptibility to a PPG excursion . Identifying this specific time window is key to trigger the timely delivery of dietary prompts, thereby increasing their relevance and behavior change potential ^36^.

To address these questions, we use a public, observational dataset of 67 adults with T2D from Shanghai, China^42^ who wore CGMs and completed self-reported logs over the course of approximately 13 days (*SD* = 1.90 days, 2,463 total PPG excursions). Building on prior work in digital biomarker development^16,43,44^ we examine: (RQ1) Can personalized ML models predict PPG excursions at the individual level? (RQ2) How does PPG prediction performance vary across individuals? We compare two models across two data collection approaches, one using passively collected, individual-level CGM observations (low-burden) and another combining CGM observations with manually logged meal and glucose-lowering agents intake (high-burden). This model comparison allows us to examine the trade-off between prediction performance and user burden with self-logging meals, a common feature in many T2D self-management apps^45–47^; thereby identifying when self-logging improves PPG predictions and when passive CGM data alone may suffice. Third, we examine (RQ3) which dietary and temporal factors best predict individual vulnerability states to PPG excursions at the individual level.

## Results

### Sample characteristics

Our final sample included 67 adults with T2D, with a mean age of approximately 61 years (*SD* = 13.39, median = 63.00), who contributed a total of 2,463 postprandial glucose (PPG) excursions over an average of approximately 13 days (*SD* = 1.90, median = 14.00). Participants reported consuming an average of 3.23 meals per day *(SD* = 0.56), totaling roughly 912.72 g of food daily (*SD* = 272.06 g), including staples (222.00 g), animal products (144.88 g), vegetables (155.93 g), fruits (31.00 g), legumes and nuts (8.81 g), dairy (82.32 g), and sweets (1.54 g). Consistent with T2D diagnostic criteria, participants had a mean HbA1c of 67.83 mmol/mol (*SD* = 26.04) and a mean fasting glucose of 157.05 mg/dl (*SD* = 59.09). On average, individuals had 36.76 usable PPG excursions (SD = 9.93), with 41.11% of those labeled as excursions—i.e., glucose values exceeding that individual’s typical postprandial baseline. Nearly all participants (94.03%) reported having occurrence of hypoglycemia. See Supplementary Table S1 for ‘Sample demographics and clinical characteristics’ and Fig. S1 for the Study Participant Flowchart.

### Personalized models predict individual PPG excursions

**Fig. 1:**
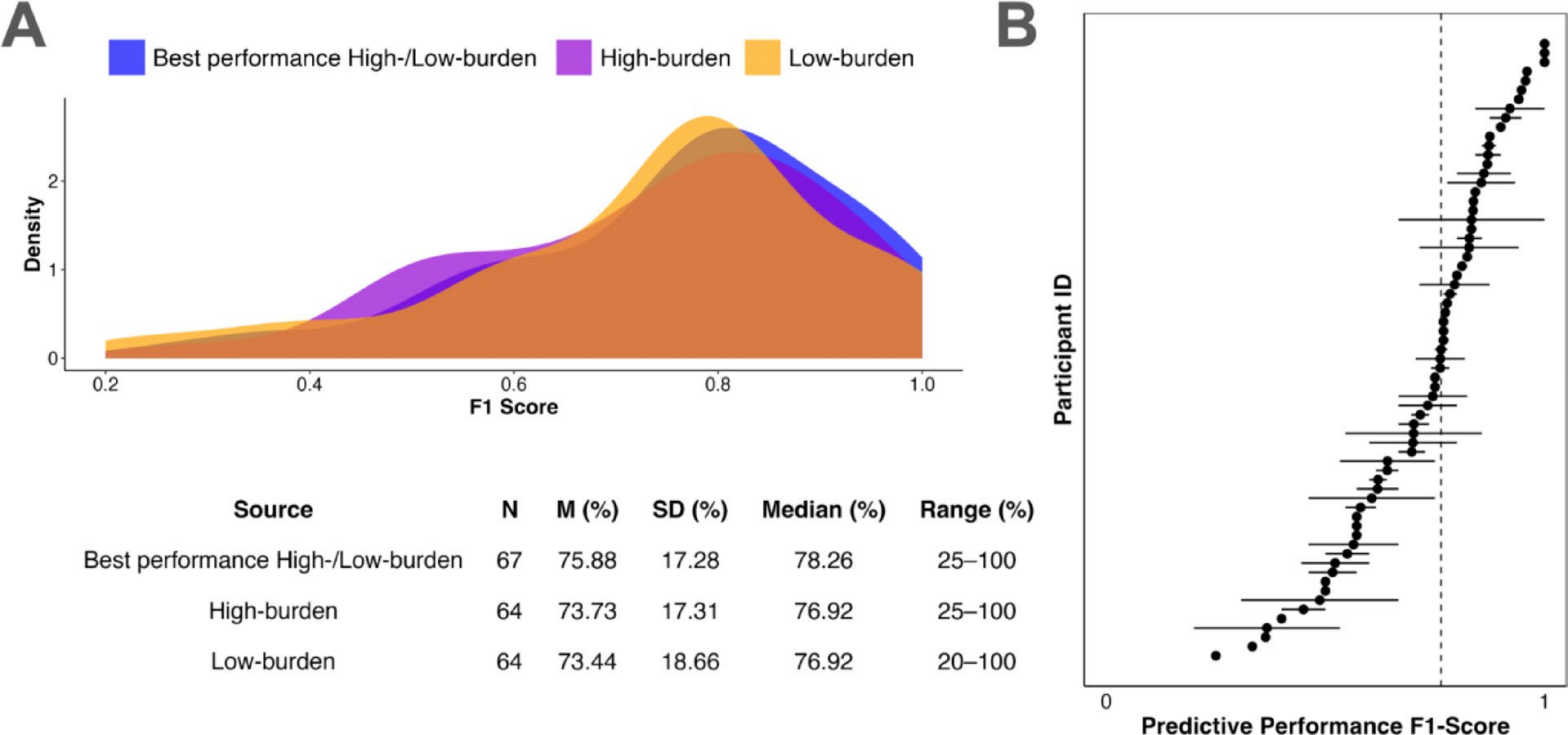
Model performance. **(A)** Performance metrics for the best personalized models (combining high-burden and low-burden models) to predict individual PPG excursions. **(B)** Inter-individual variability in model performance. Dots present the within-person median F-1 score across low-burden and high-burden models for each individual; horizontal lines show the within-person range across both low-burden and high-burden model performance for each individual; the vertical dashed line presents sample median F1-score. For three individuals, the F1-score could not be calculated for one of the two models due to undefined precision values (i.e., no positive predictions). Visualization for Fig. 1B is adapted from Refs.^48,49^.

We first examined whether personalized ML models can predict PPG excursions at the individual level. Overall, the best personalized models–created by combining the highest performing methods from the low-burden and high-burden approaches–predicted PPG excursions with a mean F1-score of 75.88% (median = 78.26%, *SD* = 17.28%). The low-burden approach, trained on the first ~ 6 days of individual-level CGM observations, achieved a mean F1-score of 73.44% (median = 76.92%, SD = 18.66%). The high-burden approach, trained on the first ~ 6 days of combined CGM observations and manual meal and medication tracking, resulted in a mean F1-score of 73.73% (median = 76.92%, *SD* = 17.31%). For more details on model performance, including precision, recall, and other metrics, see Supplementary Table S2. Inspecting the best performing F1-scores at the individual level revealed wide variability in model performance, with individual F1-scores ranging from 20 to 100% (Fig. 1B).

### Individual differences in low-vs. high-burden model performance across individuals

**Fig. 2:**
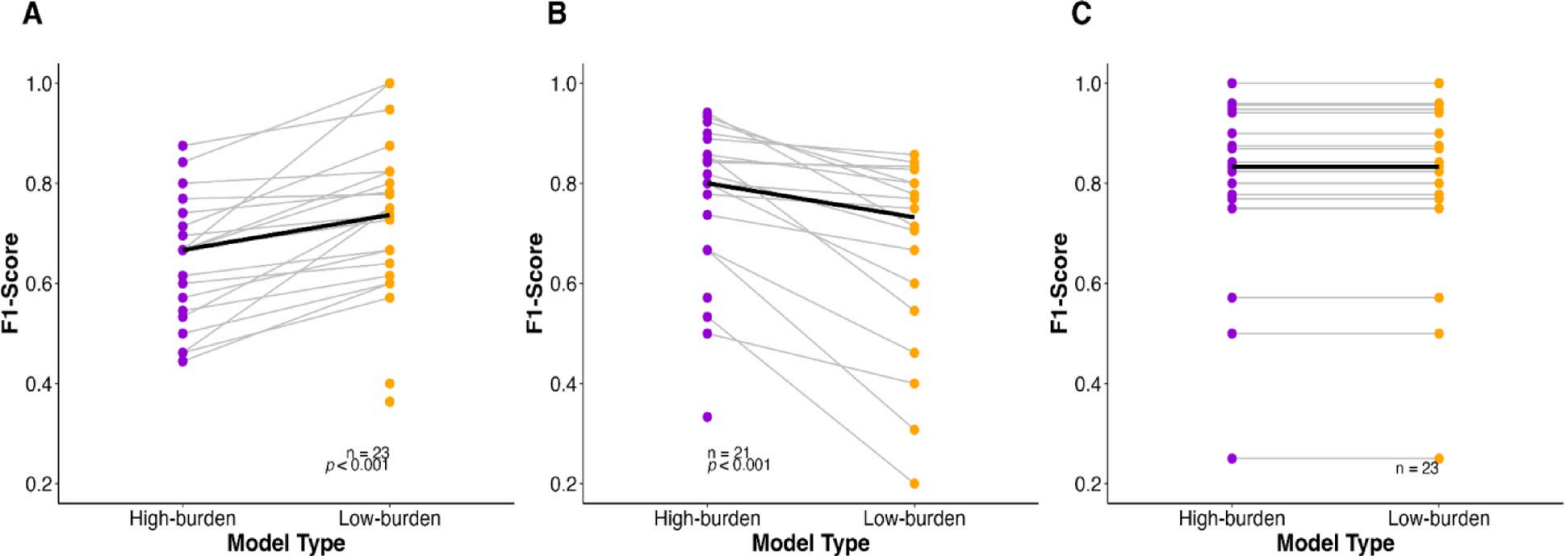
Individual differences in low-burden vs. high-burden model performance. Figures present the within-person F1-scores across both low-burden and high-burden models for (**A**) Individuals for whom the low-burden models outperformed; (**B**) Individuals for whom the high-burden models outperformed. (**C**) Individuals for whom both low-burden and high-burden models performed similarly. Statistics are based on the paired Wilcoxon signed-rank test. Individual slopes are shown by gray lines, and the median is represented by the bold black line.

Our second research question examined how PPG prediction performance varies across low-burden and high-burden models. On average, we found no differences in low-burden and high-burden model performance (Fig. 1A). However, when examining individual differences, we found that the low-burden model outperformed the high-burden model for 34.33% of the sample (*n* = 23), while the high-burden model outperformed the low-burden model for 31.34% (*n* = 21) of the sample. No differences in performance were observed for the remaining 34.33% (*n* = 23) of participants. See Fig. 2 for individual slopes across both low- and high-burden models. Wilcoxon signed-rank tests revealed that the low-burden model significantly improved the median F1-score by 7.01%, from 66.67% (high-burden model) to 73.68% (low-burden model) (Z = 4.76, *p* < 0.001, *n* = 23; Fig. 2A) for the first sub-sample. In contrast, for the second sub-sample, the high-burden model significantly increased the median F1-score by 6.79%, from 73.21% (low-burden model) to 80.00% (high-burden model) (Z = 4.48, *p* < 0.001, *n* = 21; Fig. 2B).

### Vulnerability states to PPG excursions vary widely across individuals

**Fig. 3:**
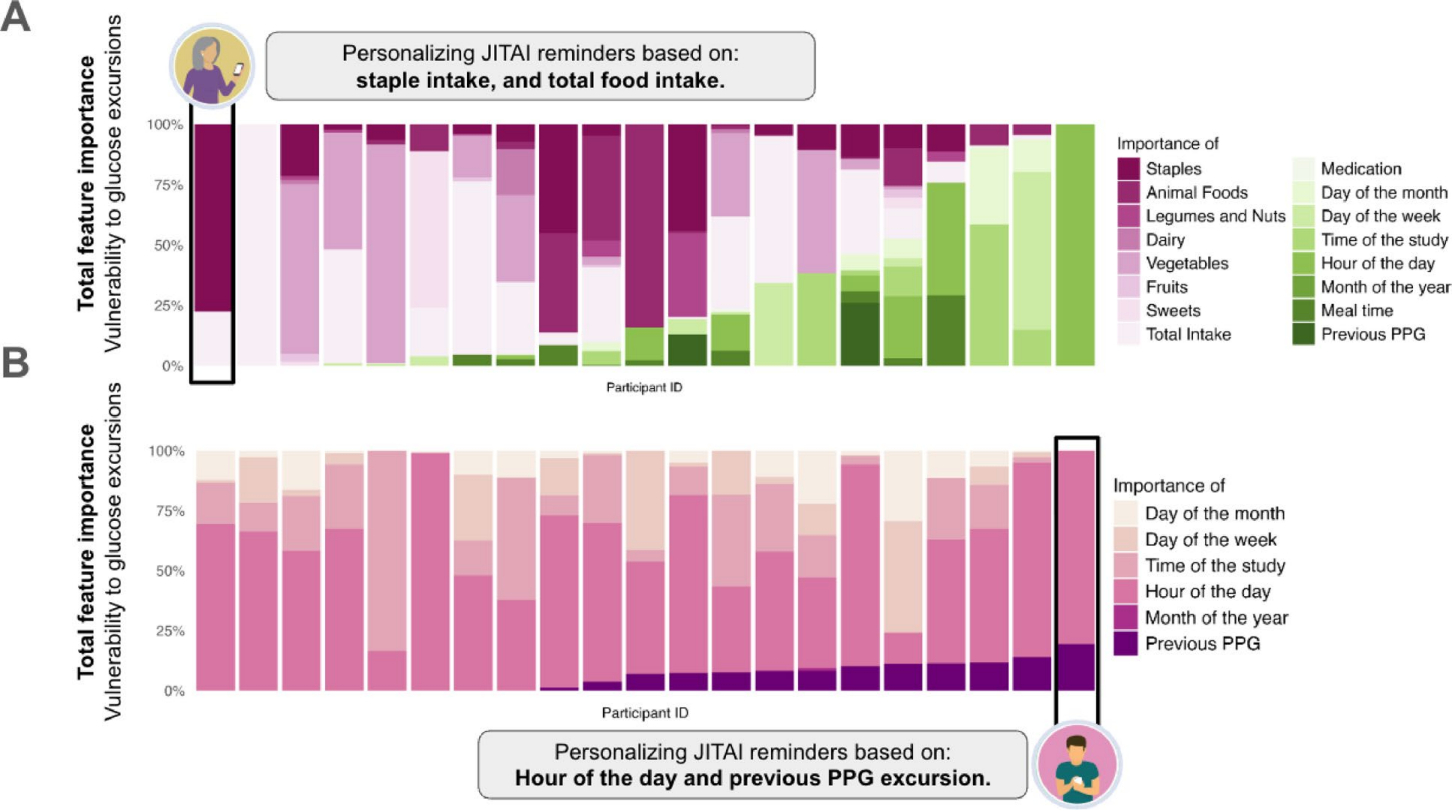
Vulnerability states predicting PPG excursions among a random subsample of participants for whom (**A**) the high-burden model or the (**B**) low-burden model performed best. The importance of each feature category (e.g., meals, temporal context) is expressed as a percentage of the total feature importance for each individual^16^.

We next investigated which dietary and temporal factors best predict individual vulnerability states to PPG excursions—defined as periods of heightened susceptibility of elevated post-meal blood glucose levels at the individual level; and how these predictors of PPG vulnerability differ across individuals. To identify the top vulnerability predictors, Following Ref.^16^, we calculated the percent contribution of each feature towards the total feature importance within each participant’s best-performing model for predicting PPG excursions. See Supplementary Table S3 for all features across high- and low-burden models. As shown in Fig. 3 and Fig. S3, no two participants shared the same PPG vulnerability state, in both direction and strength. See additional feature importance plots (Fig. S2) for individuals in whom the high-burden and low-burden models performed similarly, as well as SHAP values (Fig. S3), which further illustrate these variability patterns across models. For those for whom the high-burden model performed best (*n* = 21), the top five PPG vulnerability predictors were total grams intake, vegetable intake, staples, animal foods and hour of the day. However, none of these predictors were universally shared across participants (Fig. 3). For example, total grams intake had importance scores ranging from 0 to 100% (*M* = 23.04%) and was shared by 15 of 21 participants. Similarly, vegetable intake had an average importance of 17.05%, ranging from 0 to 90.72%, and was shared by 10 of 21 of participants. For participants for whom the low-burden performed best, the top five PPG vulnerability features included hour of the day, time in study, day of the week, day of the months, and previous PPG excursion. While the time of day was a key predictor of PPG vulnerability , the exact time when PPG excursions occurred varied widely across participants. Overall, these findings show wide inter-individual variability , highlighting that no single factor predicted vulnerability to PPG across individuals.

## Discussion

Individuals with T2D vary widely in their glycemic responses, dietary patterns, habits, and social environments, making it difficult to apply generic dietary recommendations for T2D management^22,23^. These inter-individual differences underscore the need for more effective personalized dietary interventions for T2D management^24,50,51,52^. In this study, we examined whether personalized ML models can predict PPG excursions at the individual level, a key outcome in T2D management^11^. We also examined individual differences in the predictability of PPG excursions and momentary vulnerability to these excursions, among a sample of adults with T2D in Shanghai, China; a highly relevant context as China has one of the highest T2D prevalence rates globally^53^. We found that personalized ML models can predict future PPG excursions (F1-score: *M* = 75.88%; median = 78.26%), trained on approximately six days of past individual-level CGM, dietary and temporal data. Predictive performance varied substantially across individuals, with F1-scores ranging from 20 to 100%, suggesting that the PPG excursions of some individuals were more predictable than those of others. For some individuals, predictions based solely on passively collected CGM data were sufficient. For others, predictive performance of PPG excursions improved with additional manual self-logging of meals. Notably, the contribution of meal and temporal factors in predicting PPG excursions varied widely across individuals, underscoring that there is no single, shared state of vulnerability predicting future PPG excursions.

These findings extend prior work in two key ways. First, we replicate earlier studies demonstrating that PPG responses can be feasibly predicted in real-world settings—both among healthy adults^23,54^ and adults with T1D^43,44^—using models trained on past dietary and temporal factors. Building on these findings, we show that states of vulnerability, or momentary susceptibility to PPG excursions—quantified as the relative importance of dietary or temporal factors in predicting PPG excursions—varied substantially from one participant to another. For instance, carbohydrate-rich staples (e.g., noodles, rice) were predictive of PPG excursions for 71.43% of participants (*n* = 15), with their importance ranging widely from 0 to 77.68%. Notably, these inter-individual differences were not associated with baseline variations in the amount of carbohydrates consumed per meal or hypoglycemic agent intake in our data.

Several factors may help explain the inter-individual differences in vulnerability to PPG excursions observed in our data. First, on a biological level, variation in PPG is associated with variation in individuals’ ability to digest and metabolize carbohydrates^23,54–57^. Differences in microbial composition can influence how carbohydrates are broken down and absorbed, through effects on short-chain fatty acid production, inflammation, and enzyme activity^50^. Additionally, individual variation in insulin sensitivity, exocrine pancreatic enzyme function, and glucose transporter expression may affect how efficiently glucose is cleared from the bloodstream^58^, with intestinal transit time further modulating glucose absorption^59^ and more rapid gastric emptying related to PPG excursions^60^. Gastric emptying alone is estimated to explain up to 30–40% of the variance in PPG response to carbohydrate-containing meals^61^. Further, genetic factors—such as variation in AMY1—may affect starch digestion, with those who metabolize starchy foods more rapidly potentially more likely to experience PPG excursions^57^. Together, these interconnected mechanisms may, in part, help explain why individuals with T2D experiencevariable PPG excursions, even when consuming similar meals.

In addition, contextual factors—such as macronutrient balance, food order, and meal timing—may also, in part, explain differences in vulnerability to PPG excursions^23,54,55^. For instance, even if the total carbohydrate intake is constant, meals with a higher carbohydrate ratio—more carbs relative to fat, protein, and fiber—may be more likely to produce PPG excursions. Conversely, meals with a lower carbohydrate ratio—by balancing carbohydrates with fat, protein, and fiber—may dampen PPG excursions, as these nutrients slow gastric emptying and delay the rate at which glucose enters the bloodstream^50^. Food order may also play a role, with evidence that eating carbohydrates after vegetable and/or protein-rich foods may dampen PPG excursions^62,63^. Another factor is overall meal timing, as eating late in the evening may enhance PPG excursions to the evening meal and subsequent breakfast^64^. Individuals with higher body mass index, associated with insulin resistance, may also be more vulnerable to PPG excursions, at baseline, even when consuming identical meals^56^. Overall, the inter-individual variation in PPG excursions likely reflects a complex interplay between biological predispositions and modifiable dietary behaviors—offering actionable targets for glycemic control.

Building on these insights, the state of vulnerability framework for predicting PPG excursions can be translated into digital health tools that support both patients and healthcare providers in managing T2D more effectively^65,66^. Patient-facing mobile health apps and dashboards (e.g., Oviva app^67^, mySugr^68^, and DarioHealth^69^ ) may integrate ML models that continuously track individual-level CGM data, meal logs, and daily routines to identify personalized patterns of PPG vulnerability and trigger dietary and lifestyle prompts to prevent future PPG excursions. By detecting when, and under what conditions, an individual is most likely to experience a PPG excursion—such as after eating certain food combinations or at specific times of day—these apps can deliver real-time, tailored dietary recommendations and behavioral nudges. For example, if a user’s data shows that consuming high-carbohydrate staples (e.g., white rice) in the evening consistently triggers PPG excursions, the app might send a pre-meal alert recommending lower-glycemic alternatives e.g., (cauliflower rice). Prompts could also trigger brief psychological distancing strategies, such as mindfulness exercises, to reduce cravings and create distance from high–glycemic-index cues, shown to motivate behavior change in prior work^70^ , or recommend a walk after the meal^71,72^ . Similarly, if mornings are a high-risk time for PPG excursions, prompts to choose balanced breakfast meals rich in fiber and protein can be provided adaptively. This approach—characteristic of JITAIs^36^—ensures that support is both context-aware and timely, increasing the potential of relevant behavior change.

From a healthcare provider’s perspective, personalized insights into PPG excursions can be integrated into clinical decision support systems to help enable patient-centered care and planning . Dashboards like Roche’s Diabetes Care Platform^73^ and Glooko’s Population tracker platform^74^ may integrate PPG vulnerability patterns, derived from past CGM observations and dietary logs^75^ to help providers visualize trends over time and make more informed context-based recommendations. Additionally, general practitioners (GPs), endocrinologists, and dietitians, could use visualizations of a patient’s PPG vulnerability patterns during consultations to move beyond generic dietary advice, and towards personalized meal planning and treatment based on continuous physiological and behavioral data. For instance, providers could identify specific meal components or times when a patient is most at risk and develop targeted dietary recommendations to target PPG excursions. One relevant example of embedding continuous measurements into general practice is the ongoing effort to digitize the SGED score—a measurement tool to help GPs in Switzerland monitor and improve the quality of T2D care, developed by the Swiss Society for Endocrinology and Diabetes^76^. The SGED score currently includes multiple cross-sectional indicators, such as HbA1c, blood pressure, and self-reported lifestyle measures, and is used to assess patient outcomes at both individual and population levels. Integrating continuous metrics of vulnerability to PPG excursions into the SGED score could enhance its clinical utility by capturing temporal risk patterns in patients’ daily lives. For example, signal that a patient typically experiences PPG exursions after lunch rich in refined carbohydrates—could prompt GPs to remotely adjust dietary guidance or medication timing accordingly. This kind of digital integration could also support value-based care models—which reward providers for outcomes rather than services—by incorporating short-term, dynamic indicators of glucose control that better reflect real-world patient experiences and enable timely, personalized interventions^77^. Overall, the proposed PPG vulnerability framework may help clinicians leverage granular CGM and dietary data to identify patient-specific risk patterns and adapt care plans proactively; with potential to enable more precise and proactive T2D management.

The results of this study should be interpreted in light of its strengths and limitations. A key strength is that the current observational study was conducted in a real-world setting, enhancing the ecological validity and relevance of our findings beyond the clinic or the lab. To our knowledge, this is the first study to predict individual vulnerability to PPG excursions in a Chinese adult population with T2D diabetes. However, the sample primarily comprised Chinese adults living in Shanghai who were predominantly using glucose-lowering agents, which may limit the generalizability of these results to other populations with T2D. Notably, baseline glucose profiles and PPG responses can vary by ethnicity. For example, individuals of Asian descent with T2D may have relatively higher PPG levels compared to those with Caucasian descent^78^ potentially due to differences in insulin secretion and sensitivity^79^. This variability highlights the need for caution when generalizing these findings across different populations. To improve generalizability and clinical utility, further research is needed involving more diverse demographic groups, including different ethnicities, geographic regions, treatment regimens, and larger study samples^80^. Further, incorporating additional wearables to monitor sleep, physical activity, heart rate variability, and stress, can potentially improve the predictability of PPG excursions^81^. For example, research integrating smartwatch data to track additional lifestyle measurements (e.g., sleep and stress) has shown that it is possible to improve the F1-score for glucose excursion predictions to 84–86% among healthy individuals^16^. Nevertheless, our current findings suggest that CGM and temporal data alone already provides a promising level of predictability in T2D contexts for some individuals.

One important next step is to embed the PPG vulnerability framework into Micro-Randomized Controlled Trials (mRTs)^82^, which can offer a rigorous way to evaluate the causal effects of dynamic and personalized dietary prompts for glycemic control optimized on past PPG excursions, relative to more generic standard dietary recommendations. In mRT trials, decision points—such as mealtimes—can be repeatedly randomized to either deliver or withhold tailored or personalized prompts (e.g., a food swap suggestion or a motivational nudge based on prior PPG excursions). This intervention design enables researchers to identify which dietary recommendations may work best, for whom, and under what circumstances at the individual level^83^. Crucially, this step also requires distinguishing between within-person and between-person variability in PPG excursions. Some predictors of PPG vulnerability may reflect relatively stable, between-person characteristics, such as microbiome composition, insulin sensitivity, or genetic variation. Others may reflect dynamic, within-person states that fluctuate over time—such as momentary stress, previous night sleep, physical activity in the past hour, or prior snack intake. Disentangling these sources of variation is essential for adaptive JITAI design, as it enables ML models to adjust not only between individuals but also within the same individual across changing temporal contexts^84^. Future longitudinal mRT studies can map these evolving factors and inform the timing, content, and intensity of personalized dietary interventions targeting PPG excursions among individuals with T2D.

In conclusion, we found that personalized ML models can feasibly predict individual PPG excursions among a sample of Chinese adults with T2D who are undertaking glucose-lowering agents; with individual predictors to PPG excursions varying widely across individuals. No two participants shared the same state of vulnerability to PPG excursions. Future research can integrate these individual-level PPG vulnerability patterns in JITAIs, mHealth apps, and clinical decision support tools to personalize context-aware dietary interventions, with potential to optimize more effective, patient-centered diabetes care .

## Methods

### Data

We used the ShanghaiT2DM Dataset^42^ a publicly available, observational dataset of adults with T2D, recruited in Shanghai, China. The dataset, made available by Zhao and colleagues^85^ was developed to support ML research aimed at improving glycemic control. Participants were enrolled through the Diabetes Data Registry and Individualized Lifestyle Intervention at Shanghai East Hospital (September 2019 to March 2021) and Shanghai Fourth People’s Hospital (June 2021 to November 2021). Our study made use of the complete ShanghaiT2DM dataset, version 2, released on January 4, 2023^85^. Given that the current investigation presents a secondary analysis of an existing dataset, no additional recruitment was conducted beyond the original data collection period (September 2019 to November 2021). The current investigation was conducted using publicly available, anonymized data,^85^, and thus ethics approval is not applicable. The original data collection and methods were approved by the Ethics Committees of Shanghai East Hospital and Shanghai Fourth People’s Hospital, affiliated with Tongji University, in accordance with the Declaration of Helsinki. Informed consent was obtained from all participants. The original study ethics approval number is not provided in the public documentation for secondary analyses.

### Participants

Eligible participants included 109 patients diagnosed with T2D^86^ aged 18 or older, who had available CGM recordings for at least 3 days. Patients were excluded if they reported alcohol or drug abuse, were unable to comply with the study, or were deemed unsuitable by the original investigators who completed the data collection. Our analysis included 67 participants with T2D, with a total 2’463 PPG observations. Forty-two participants were excluded from our analyses (from these: *n* = 1 for having no available PPG observations; *n* = 36 for having insufficient PPG observations to split the data in a train-test split; ≤20 observations in total; and *n* = 5 due to insufficient variability to run the predictive models, with all observations belonging to the same class; see Supplementary Fig. S1 ‘Study flowchart and exclusion criteria’).

### Study procedure

Participants were instructed to wear a CGM device, FreeStyle Libre (Abbott Diabetes Care^87^), and to self-report their meal and glucose-lowering agent intake over the course of 14 days. Participants also provided laboratory blood measurements, beyond the scope of the current investigation, six months prior to the observational data collection.

### Measures

Data for model building included four types of features: CGM recordings, self-reported meal content, glucose-lowering agents intake, and temporal measures created through time stamps. See Supplementary Table S3 ‘Features categorization’ for the full feature list and categorization.

#### Meal content features

The meal logs provided by participants were categorized into food groups commonly consumed in China^88^ including: (1) staples (e.g., rice, noodles), (2) vegetables, (3) fruits, (4) animal foods (e.g., chicken, eggs), (5) dairy products, (6) legumes, nuts, and seeds, and (7) sweets. These food groups were coded as both binary variables and as food group grams per meal, in addition to the total grams of food intake. Consistent with prior work^16^ all meal content and glucose-lowering agent intake variables were calculated for the current period and lagged for the previous 8-hour intake and the previous 24-hour intake.

#### Glucose-lowering agent intake features

Participants recorded their daily glucose-lowering agent intake including the exact timing. Medication logs were converted into time-varying variables to account for the different effects and doses of diabetes insulin medications on blood sugar levels over time. Basal and intermediate-acting insulins have a gradual effect over 2 to 24 h^89,90^; bolus insulin acts quickly within 15 min to 4 hours^91^; and non-insulin hypoglycemic agents start working immediately and last for about 6 hours^92^.

#### Temporal features

Temporal features were created from the time stamps in the meal survey logs. Variables were created for mealtime, day of the month, months of the year, hour of the day and day of the study duration. To account for possible fasting periods, binary mealtime indications in the last 8 h and 24 h, day of the week, were also included.

### PPG excursions

Based on previous work^16^, we calculated PPG excursions, relative to an individuals’ PPG baseline, as our main outcome. A binary variable for 2-hour PPG was determined after each meal, indicating whether it was higher or lower than the previous mean PPG observation, thus providing each participant with a personal rolling baseline. The 2-hour PPG value was calculated using the 2-hour incremental area under the curve following a meal^93^. This allowed us to compute PPG excursion approximately 2 h after each meal relative to each participant’s previous value^16^.

### Model development

#### Data preparation

We developed two personalized ML models for each participant, a low-burden and a high-burden model. The low-burden model included the following features: previous (lagged) PPG excursion and temporal features (such as mealtime, time of day). The high-burden-model included the same features, in addition to dietary intake (food groups and total intake per meal, in the last 8 and 24 h) and self-reported diabetes medicine-intake (glucose-lowering agents including non-insulin hypoglycemic agents and bolus, basal, and intermediate insulin intake, dose, and effect time). See Supplementary Table S3 ‘Features categorization’ for full features and their categorization. Missing values were imputed using single imputation, and missingness dummy variable columns were introduced in the model.

#### Model training and cross-validation

For each individual, we employed a 70/30 within-person training-test split due to the time series nature of the data. The first 70% of the observations served as the training set, while the remaining 30% was used as the test set. To determine the final model, we performed time series rolling origin cross-validation (CV) with four folds on the training set. Rolling origin cross-validation involves sequentially expanding the training data window while keeping the test data fixed. This approach is specific to time-series data and ensures that the model does not use future information to predict past events^49,94^.

To enable a train-test split (requiring at least 14 observations for training and 6 for testing) and further subdivision of the training set into a 4-fold rolling origin cross-validation scheme, we required a minimum of 20 PPG observations per individual. Participants with fewer than 20 observations were excluded from the analysis to ensure sufficient data for both model training and validation. This threshold was selected to balance the trade-off between maximizing data inclusion and maintaining the integrity of the time-series modeling approach, in line with prior studies^80,95,96^. As the current study is a secondary analysis of a publicly available dataset, no *a-priori power* calculation was conducted. We used all available data provided by the original authors. Although power calculation in machine learning or emerging person-specific approaches are often not considered^97^ the sample size used in the prior analyses is comparable to prior studies developing similar predictive personalized, predictive models to allow for robust estimates (see example Refs^98,99^).

#### Hyper-parameter tuning

Hyperparameters for XGBoost were determined using a 4-fold CV grid search method (nrounds = seq(from = 50, to = 150, by = 50); eta = c(0, 0.0001, 0.001, 0.01, 0.015); max_depth = c(1, 2, 3); gamma = c(−0.5, 0, 0.5); colsample_bytree = c(0.5, 1, 2); min_child_weight = c(1, 2); subsample = 1), which systematically explores various combinations to find the optimal settings based on predefined criteria.

#### Model evaluation

To predict PPG excursions, we employed personalized Extreme Gradient Boosting (XGBoost)^100^ a composite learning algorithm used in similar, prior work^101^. To evaluate model performance (RQ1), we used F1-score, which balances precision and recall in classifying PPG excursions^102^. To compare the performance of low-burden and high-burden models (RQ2), we compared the model performance F-scores using Wilcoxon signed-rank tests, to account for non-normality.

#### Feature importance

To determine feature importance for each individual model, we utilized gain-based global feature importance and SHAP (SHapley Additive exPlanations) values derived from XGBoost^103^.

#### PPG vulnerability

To assess each individual’s vulnerability to PPG excursions (RQ3), we computed gain-based global feature importance to quantify the contribution of each feature to the model’s predictions. For each individual, we calculated the mean absolute importance value for each feature across all data points, representing its average impact on the model’s output. To express these contributions as relative percentages, we normalized each feature’s mean absolute importance value by dividing it by the sum of all features’ mean absolute importance values for that individual. This normalization yielded percentage contributions, enabling us to rank features based on their relative impact on the model’s predictions for each participant. The resulting pattern of relative feature importances constitutes the individual’s PPG vulnerability profile, highlighting which factors most significantly influence their glycemic responses. All analyses were conducted using R version 4.4.1.

## Electronic supplementary material

Below is the link to the electronic supplementary material.

Supplementary Material 1

## Data Availability

The original dataset analyzed during the current study is available in the Figshare repository by Zhao et al. 2023: https://figshare.com/collections/Diabetes_Datasets_ShanghaiT1DM_and_ShanghaiT2DM/6310860/2.

## Code availability

The code and data to reproduce the data pre-processing and analyses for the given study are available at the following repository: https://github.com/VictoriaKat/Scientific-Reports-T2D-Glucose-Excursions.
