# Supplementary material for: Predicting postprandial glucose excursions to personalize dietary interventions for type-2 diabetes management
Source: Sci Rep. 2025 Jul 17;15:25920. doi: 10.1038/s41598-025-08003-4 (PMC12271334; doi:10.1038/s41598-025-08003-4)
Supplement: Supplementary file 1 — Supplementary Material 1 [file 41598_2025_8003_MOESM1_ESM.docx]

**Supplementary materials**

**Table S1.|** Sample demographics and clinical characteristics.

|  | Overall  sample | High-burden  model subset | Low-burden  Model subset | Equal model  Performance subset |
| --- | --- | --- | --- | --- |
| *N* | 67 | 21 | 23 | 23 |
| F1 Score | *0.78 [0.67, 0.88]* | *0.80 [0.67, 0.86]* | *0.74 [0.61, 0.81]* | *0.83 [0.77, 0.94]* |
| Gender = Male (%) | *32 (47.8)* | *8 (38.1)* | *12 (52.2)* | *12 (52.2)* |
| Age [Years] (mean (SD)) | *61.39 (13.39)* | *59.43 (14.08)* | *63.30 (10.19)* | *61.26 (15.70)* |
| Duration of Diabetes [Years] (median [IQR]) | 7.00 [1.00, 15.50] | 5.00 [1.00, 15.00] | 8.00 [1.75, 15.50] | 7.00 [0.79, 15.00] |
| Fasting Plasma Glucose [mg/dL] (median [IQR]) | 149.40 [113.40, 172.80] | 144.36 [104.40, 165.60] | 137.70 [117.14, 166.95] | 171.36 [125.19, 198.45] |
| HbA1c [mmol/mol] (median [IQR]) | 59.57 [50.82, 78.15] | 56.29 [43.17, 68.86] | 59.57 [52.19, 64.48] | 71.04 [56.02, 109.84] |
| BMI (median [IQR]) | 23.60 [22.14, 25.94] | 24.39 [22.22, 26.23] | 22.84 [21.90, 24.28] | 24.62 [22.37, 26.34] |
| Mean Postprandial* [mg/dL] (median [IQR]) | 227.16 [180.54, 291.60] | 201.78 [167.40, 228.96] | 212.58 [164.52, 311.94] | 270.00 [225.54, 321.75] |
| Duration [day] (median [IQR]) | 14.00 [12.00, 14.00] | 14.00 [12.00, 14.00] | 14.00 [13.00, 14.00] | 13.00 [12.00, 14.00] |
| Observations/Meals [per day] (median [IQR]) | 3.07 [2.85, 3.52] | 3.29 [2.94, 3.57] | 3.27 [2.85, 3.84] | 2.93 [2.83, 3.10] |
| Staples [grams per day] (median [IQR]) | 197.77 [132.21, 299.26] | 218.67 [134.07, 301.86] | 198.08 [143.54, 248.66] | 171.82 [118.65, 305.45] |
| Vegetables [grams per day] (median [IQR]) | 133.93 [87.99, 219.06] | 151.00 [116.43, 211.43] | 160.00 [87.99, 225.96] | 123.46 [85.19, 192.49] |
| Fruits [grams per day] (median [IQR]) | 5.83 [0.00, 32.28] | 12.62 [4.00, 36.43] | 5.83 [0.00, 40.14] | 0.08 [0.00, 12.50] |
| Animal Foods [grams per day] (median [IQR]) | 137.93 [104.24, 187.32] | 140.43 [110.30, 179.07] | 132.50 [97.50, 161.77] | 142.71 [99.28, 201.42] |
| Dairy Products [grams per day] (median [IQR]) | 57.33 [0.00, 132.25] | 55.00 [0.00, 101.67] | 80.77 [0.00, 174.04] | 46.15 [0.00, 144.75] |
| Legumes Nuts [grams per day] (median [IQR]) | 4.00 [0.00, 14.82] | 5.00 [0.00, 15.71] | 3.57 [0.00, 10.66] | 3.85 [0.00, 15.82] |
| Sweets [grams per day] (median [IQR]) | 0.00 [0.00, 0.00] | 0.00 [0.00, 1.29] | 0.00 [0.00, 0.00] | 0.00 [0.00, 0.00] |
| Total Food [grams per day] (median [IQR]) | 885.64 [704.42, 1,117.15] | 908.21 [721.15, 1,165.64] | 885.64 [709.14, 1,030.22] | 822.54 [705.93, 1,117.15] |
| SD of Meal Timing  [min] |  |  |  |  |
| Morning (5 am–10 am) | 33.28 [25.81, 54.43] | 40.5 [28.13, 59.23] | 40 [27.57, 54.21] | 31.05 [21.62, 43.55] |
| Lunch (10 am–2 pm) | 32.07 [24.49, 44.08] | 30.9 [26.31, 44.64] | 32.07 [25.48, 43.31] | 32.36 [22.25, 43] |
| Afternoon (2 pm–6 pm) | 26.83 [0, 63.43] | 53.67 [0,60] | 30 [18.09, 76.65] | 26.46 [0, 42.43] |
| Dinner (6 pm–9 pm) | 31.13 [0, 42.43] | 28.34 [0, 48.68] | 31.26 [0, 34.65] | 36.18 [9.05, 44.21] |

**Note.** High-burden subset includes individuals for whom the high-burden model performed best; the low-burden subset included individuals for whom the low-burden model performed best. Test statistics are based on Kruskal-Wallis Rank Sum test. *Mean Postprandial glucose laboratory measurement measured in clinical setting.

**
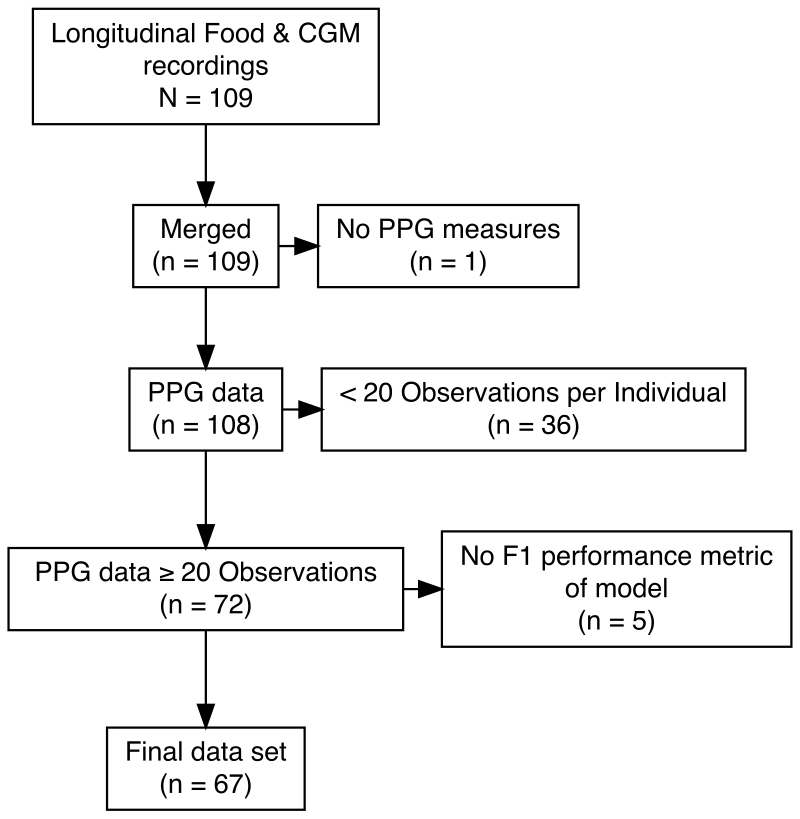
**

**Fig S1.|** Study flowchart and exclusion criteria.

Flowchart for participants data exclusion, with the final sample including 2463 glucose observations. No F1 performance due to undefined precision values (i.e., no positive predictions).

**Table S2.|** Model performance metrics.

|  | **Accuracy (%)** | **Recall (%)** | **Precision (%)** | **F1 Score (%)** | **AUC (%)** |
| --- | --- | --- | --- | --- | --- |
| **High-burden model** |  |  |  |  |  |
| *Mean* | 67.39 | 76.87 | 73.10 | 73.73 | 62.58 |
| *SD* | 17.76 | 28.15 | 21.91 | 17.31 | 18.64 |
| *Median* | 69.23 | 87.50 | 75.00 | 76.92 | 62.50 |
| **Low-burden model** |  |  |  |  |  |
| *Mean* | 68.02 | 76..95 | 73.83 | 73.44 | 63.48 |
| *SD* | 17.99 | 29.54 | 20.20 | 18.66 | 18.63 |
| *Median* | 70.00 | 87.50 | 71.43 | 76.92 | 63.10 |
| **Best performance High-/Low-burden model** |  |  |  |  |  |
| *Mean* | 71.59 | 84.55 | 74.06 | 75.88 | 65.27 |
| *SD* | 16.62 | 22.82 | 18.64 | 17.28 | 19.39 |
| *Median* | 72.73 | 100 | 71.43 | 78.26 | 70.00 |

**Note.** High-burden model includes manual meal-content features and medicine intake features, in addition to the CGM, temporal features. Low-burden model includes CGM and temporal features.

**
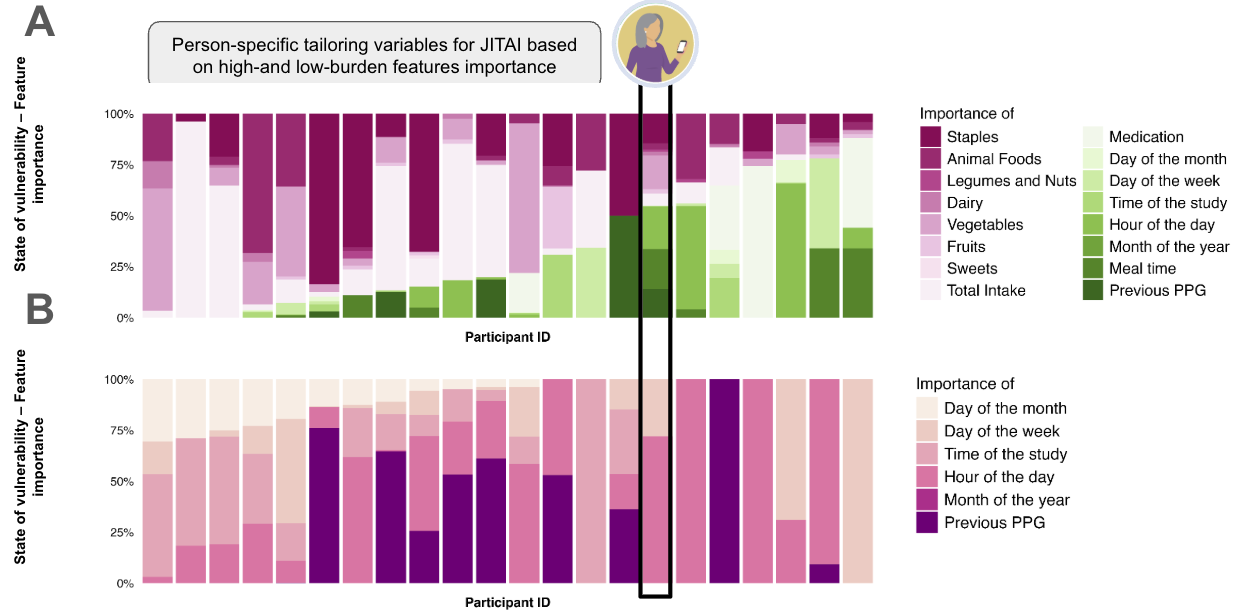
**

**Fig S2. |** Feature importance predicting postprandial glucose excursions among a subset of participants where (A) the high-burden and (B) the low-burden models showed equal performance. One individual for whom feature importance could not be calculated in the low-burden model was excluded in both plots. Visualizations showcase the diversity in vulnerability predictors across individuals.

**
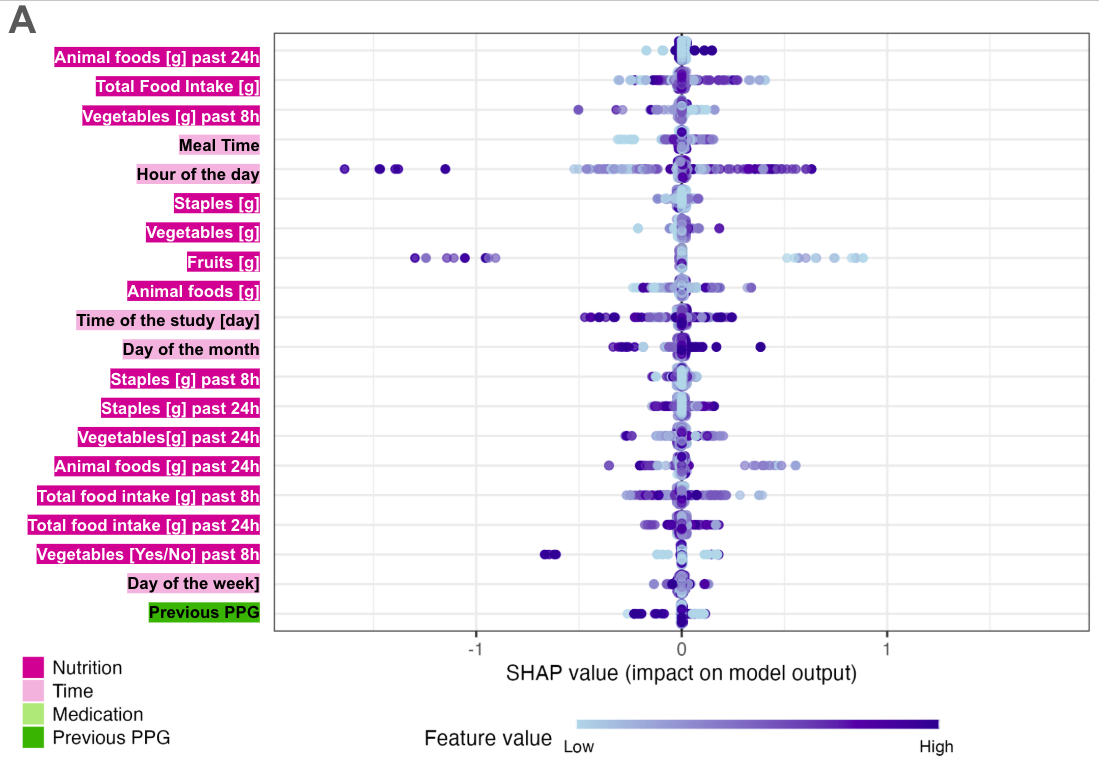

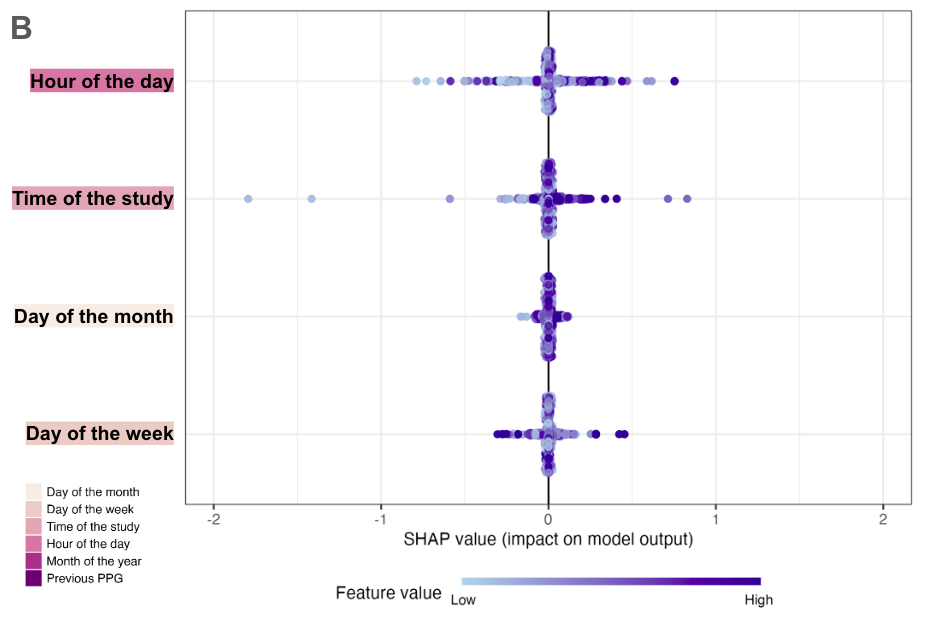
**

**Fig S3.|** SHAP value contribution of each feature based on (A) high-burden model with the 20 most important features and (B) low-burden model with the 4 most important features, showcasing the diversity in the strength and direction of the predictors across both models.

**Table S3.|** Features categorization

| **Feature** | **Category** | **Nutrition Category** | **Model** |
| --- | --- | --- | --- |
| **Staples_g_24h** | Nutrition | Staples | High-burden |
| **Animal_foods_bin_8h** | Nutrition | Animal Foods | High-burden |
| **Fruits_bin_24h** | Nutrition | Fruits | High-burden |
| **Fruits_bin_8h** | Nutrition | Fruits | High-burden |
| **Vegetables_bin_24h** | Nutrition | Vegetables | High-burden |
| **Vegetables_bin_8h** | Nutrition | Vegetables | High-burden |
| **Staples_bin_24h** | Nutrition | Staples | High-burden |
| **Staples_bin_8h** | Nutrition | Staples | High-burden |
| **Total_g_24h** | Nutrition | Total Intake | High-burden |
| **Total_g_8h** | Nutrition | Total Intake | High-burden |
| **Animal_foods_bin_24h** | Nutrition | Animal Foods | High-burden |
| **Staples_g_8h** | Nutrition | Staples | High-burden |
| **Vegetables_g_8h** | Nutrition | Vegetables | High-burden |
| **Vegetables_g_24h** | Nutrition | Vegetables | High-burden |
| **Fruits_g_8h** | Nutrition | Fruits | High-burden |
| **Fruits_g_24h** | Nutrition | Fruits | High-burden |
| **Animal_foods_g_8h** | Nutrition | Animal Foods | High-burden |
| **Animal_foods_g_24h** | Nutrition | Animal Foods | High-burden |
| **Dairy_Products_g_8h** | Nutrition | Dairy | High-burden |
| **Dairy_Products_g_24h** | Nutrition | Dairy | High-burden |
| **Legumes_Nuts_g_8h** | Nutrition | Legumes and Nuts | High-burden |
| **Sweets_g_24h** | Nutrition | Sweets | High-burden |
| **Legumes_Nuts_g_24h** | Nutrition | Legumes and Nuts | High-burden |
| **Dairy_Products_bin_8h** | Nutrition | Dairy | High-burden |
| **Legumes_Nuts_bin_8h** | Nutrition | Legumes and Nuts | High-burden |
| **Sweets_bin_24h** | Nutrition | Sweets | High-burden |
| **Sweets_bin_8h** | Nutrition | Sweets | High-burden |
| **Legumes_Nuts_bin_24h** | Nutrition | Legumes and Nuts | High-burden |
| **Staples_g** | Nutrition | Staples | High-burden |
| **Staples_bin** | Nutrition | Staples | High-burden |
| **Vegetables_g** | Nutrition | Vegetables | High-burden |
| **Vegetables_bin** | Nutrition | Vegetables | High-burden |
| **Dairy_Products_bin_24h** | Nutrition | Dairy | High-burden |
| **Fruits_bin** | Nutrition | Fruits | High-burden |
| **Fruits_g** | Nutrition | Fruits | High-burden |
| **Animal_foods_bin** | Nutrition | Animal Foods | High-burden |
| **Dairy_Products_g** | Nutrition | Dairy | High-burden |
| **Dairy_Products_bin** | Nutrition | Dairy | High-burden |
| **Legumes_Nuts_g** | Nutrition | Legumes and Nuts | High-burden |
| **Legumes_Nuts_bin** | Nutrition | Legumes and Nuts | High-burden |
| **Table S3. Continued \|** Features categorization | | | |
| **Sweets_g** | Nutrition | Sweets | High-burden |
| **Sweets_bin** | Nutrition | Sweets | High-burden |
| **total_g** | Nutrition | Total Intake | High-burden |
| **Animal_foods_g** | Nutrition | Animal Foods | High-burden |
| **Sweets_g_8h** | Nutrition | Sweets | High-burden |
| **Meal_bin_8h** | Meal time |  | High-burden |
| **Meal_bin_24h** | Meal time |  | High-burden |
| **month** | Month of year |  | High- & low-burden |
| **Hour** | Hour of day |  | High- & low-burden |
| **Day** | Day of study duration |  | High- & low-burden |
| **weekday_num** | Day of week |  | High- & low-burden |
| **Meal_time** | Meal time |  | High-burden |
| **day** | Day of month |  | High- & low-burden |
| **Non_Insulin_Time** | Medication |  | High-burden |
| **Bolus_insulin_Time** | Medication |  | High-burden |
| **Intermediate_insulin_Time** | Medication |  | High-burden |
| **Insulin_Basal_Time** | Medication |  | High-burden |
| **basal_insulin_dose_time** | Medication |  | High-burden |
| **bolus_insulin_dose** | Medication |  | High-burden |
| **basal_insulin_dose** | Medication |  | High-burden |
| **intermediate_insulin_dose_time** | Medication |  | High-burden |
| **Intermediate_insulin** | Medication |  | High-burden |
| **bolus_insulin** | Medication |  | High-burden |
| **basal_insulin** | Medication |  | High-burden |
| **Non.insulin.hypoglycemic.agents** | Medication |  | High-burden |
| **bolus_insulin_dose_time** | Medication |  | High-burden |
| **intermediate_insulin_dose** | Medication |  | High-burden |
| **PPG_spike_prev** | Previous PPG | | High- & low-burden |

**Note.** “High-burden” indicates that only high-burden features were used in the model. “High- & low-burden” indicates that both high- and low-burden features were included.
